# Multi-Omics Profiling Reveals Glycerolipid Metabolism-Associated Molecular Subtypes and Identifies ALDH2 as a Prognostic Biomarker in Pancreatic Cancer

**DOI:** 10.3390/metabo15030207

**Published:** 2025-03-18

**Authors:** Jifeng Liu, Shurong Ma, Dawei Deng, Yao Yang, Junchen Li, Yunshu Zhang, Peiyuan Yin, Dong Shang

**Affiliations:** 1 Department of General Surgery, The First Affiliated Hospital of Dalian Medical University, Dalian 116000, China; liujf01@dmu.edu.cn (J.L.); mashurong@dmu.edu.cn (S.M.); yangy39@dmu.edu.cn (Y.Y.); lijc03@dmu.edu.cn (J.L.); 2 Clinical Laboratory of Integrative Medicine, The First Affiliated Hospital of Dalian Medical University, Dalian 116000, China; zhangys01@dmu.edu.cn; 3 Department of Hepato-Biliary-Pancreas, Affiliated Hospital of North Sichuan Medical College, Nanchong 637000, China; ddwtougao@163.com; 4 Institute of Integrative Medicine, Dalian Medical University, Dalian 116000, China

**Keywords:** pancreatic cancer, glycerolipid metabolism, molecular classification, multi-omics technologies, ALDH2

## Abstract

**Background**: The reprogramming of lipid metabolism, especially glycerolipid metabolism (GLM), plays a key role in cancer progression and response to therapy. However, the role and molecular characterization of GLM in pancreatic cancer (PC) remain unclear. **Methods**: A pan-cancer analysis of glycerolipid metabolism-related genes (GMRGs) was first conducted to assess copy-number variants, single-nucleotide variations, methylation, and mRNA expression. Subsequently, GLM in PC was characterized using lipidomics, single-cell RNA sequencing (scRNA-seq), and spatial transcriptomic analysis. A cluster analysis based on bulk RNA sequencing data from 930 PC samples identified GLM-associated subtypes, which were then analyzed for differences in prognosis, biological function, immune microenvironment, and drug sensitivity. To prioritize prognostically relevant GMRGs in PC, we employed a random forest (RF) algorithm to rank their importance across 930 PC samples. Finally, the key biomarker of PC was validated using PCR and immunohistochemistry. **Results**: Pan-cancer analysis identified molecular features of GMRGs in cancers, while scRNA-seq, spatial transcriptomics, and lipidomics highlighted GLM heterogeneity in PC. Two GLM-associated subtypes with significant prognostic, biofunctional, immune microenvironmental, and drug sensitivity differences were identified in 930 PC samples. Finally, ALDH2 was identified as a novel prognostic biomarker in PC and validated in a large number of datasets and clinical samples. **Conclusions**: This study highlights the crucial role of GLM in PC and defines a new PC subtype and prognostic biomarker. These findings establish a novel avenue for studying prognostic prediction and precision medicine in PC patients.

## 1. Introduction

Pancreatic cancer (PC) is an extremely aggressive gastrointestinal malignancy with a grim prognosis [1]. PC has become the third leading cause of cancer death [2], highlighting the urgent need for the development of new therapeutic strategies. Despite significant advances, PC remains a highly complex and heterogeneous disease, making molecular classification a promising approach to optimize prognostic prediction and provide precision medicine for individual patients [3]. Large-scale studies have explored the somatic genome in PC at the genomic and transcriptomic levels, aiming to identify novel therapeutic targets. However, current therapeutic strategies targeting genomic alterations have only shown success in 8% to 15% of patients with defined driver mutations in the epithelial compartment [4,5,6]. Recent advances in multi-omics technologies, such as RNA sequencing, proteomics, and epigenetic profiling, have improved our understanding of PC. These tools have enabled molecular classifications based on histopathology, mutations, and transcriptomics. However, these classifications often overlook the crucial role of metabolic processes in prognosis and therapeutic response.

Metabolic reprogramming is increasingly recognized as a key enabling hallmark of cancer, facilitating tumor progression through dynamic nutrient acquisition and energy adaptation [7,8,9]. Among various metabolic pathways, lipid metabolism has gained increasing attention due to its critical role in cancer biology [10,11]. Lipids, key components of cellular membranes and signaling pathways, play a vital role in cancer cell survival and proliferation [12]. Currently, a large body of evidence suggests that abnormalities in lipid metabolism, including membrane formation, lipid synthesis and degradation, and lipid-driven cell signaling, are present in various cancers [13,14,15]. Among them, glycerol-based lipids play a crucial role in human physiology and disease, ranging from disorders of fat storage and metabolism to tumor cell survival and proliferation [16]. The accumulation of lipid droplets in non-adipocytes is a prominent feature of cancer, and its core component, triacylglycerol (TAG), may contribute to tumorigenesis and progression by supporting energy homeostasis and combating cellular stress [11,17]. Diacylglycerol (DAG) is broadly implicated in tumorigenesis, progression, and metastasis. The DAG pathway is capable of shaping tumor ecosystems by regulating the complex and dynamic interactions between cancer cells and the immune landscape, which has great potential in targeting cancer therapies [18]. In addition, monoacylglycerols (MAGs) are now considered an important group of active signaling molecules, and differences in their levels have been found to be relevant in many cancer studies [19,20,21]. Furthermore, the intricate interplay between glycerolipids and other lipids highlights the need for a deeper understanding of the glycerolipid metabolism (GLM) network. GLM characterization may uncover novel strategies for early diagnosis, prognostic assessment, and personalized treatment in PC patients.

In this study, we summarized the genetic characteristics of glycerolipid metabolism-related genes (GMRGs) in human malignant tumors for the first time. The potential significance of GLM in PC was explored at the single-cell level, and the activities of GLM in different cellular subpopulations of PC were revealed. In addition, lipidomic analysis of clinical serum samples further confirmed the presence of significantly abnormal GLM in PC. Subsequently, two GLM-related molecular subtypes were identified that were associated with the prognosis of PC, the tumor immune microenvironment (TIME), and the therapeutic response. Finally, based on the random forest (RF) algorithm, aldehyde dehydrogenase 2 (ALDH2) was identified as the GMRG most associated with PC prognosis, which was validated in ten datasets from the BEST website and 62 clinical tissue samples. In conclusion, this study characterizes the GLM features of PC with a comprehensive multi-omics analysis and suggests new targets and strategies for the stratification and precision treatment of PC patients.

## 2. Materials and Methods

### 2.1. Study Scheme

First, a pan-cancer analysis of GMRGs was performed to understand their molecular features in different cancer types. Single-cell RNA sequencing (scRNA-seq) and spatial transcriptomic analysis were utilized to assess GLM levels in PCs. Subsequent lipidomic analysis further revealed significant GLM dysregulation in PC. Finally, the molecular subtypes and biomarkers in PC were identified using transcriptomic data to study prognostic prediction and precision medicine in PC patients.

### 2.2. Pan-Cancer Analysis of GMRGs

The GMRGs (KEGG_GLYCEROLIPID_METABOLISM) were obtained from the Molecular Signatures Database (MSigDB, http://www.gsea-msigdb.org/ (accessed on 24 November 2024)). Copy number variation (CNV), single-nucleotide variation (SNV), methylation, transcript, and clinical data of various cancers were downloaded from TCGA to investigate the pan-cancer genetic characteristics of the GMRGs [22]. The “limma” package in R was used to analyze the differential expression of GMRGs in tumor and peritumoral tissues [23]. The expression profiles were combined with survival information using Cox regression to explore the prognostic significance of GMRGs. The results of all analyses are presented as heatmaps.

### 2.3. scRNA-seq and Spatial Transcriptome Data Analysis

scRNA-seq data were collected from the CRA001160 dataset [24]. The data were first filtered according to previous criteria and visualized using the “VlnPlot” function [25]. The data were then normalized, scaled, and processed using the “sctransform” function. The “Harmony” function was used to eliminate batch effects. The “FindNeighbors” and “FindClusters” functions were used to assess the similarity between cells and clustered cell subpopulations. We used the “FindAllMarkers” method to identify specific expression markers for each cell subtype and annotate cells based on the characteristics of known markers [24]. In addition, “CopyKat” was used to determine whether a cell was malignant with an aneuploid chromosome or normal with a diploid chromosome by inferring chromosome copy number changes [26]. We comprehensively evaluated each cell’s GLM signaling score using six scRNA-seq gene set scoring methods: AddModuleScore [27], AUCell [28], UCell [29], Singscore [30], ssgsea [31], and total score.

In addition, the spatial transcriptomic data of the three PC samples were derived from the GSE111672 dataset [32]. We utilized the “Read10X_Image” function to retrieve spatial cell localization information. We similarly utilized the six gene set-scoring algorithms described above for a comprehensive assessment of GLM signaling in the spatial context of PC.

### 2.4. Lipidomic Analysis for Serum Samples in PC Patients

Peripheral blood samples were collected from PC patients (n = 18) and healthy controls (n = 32) at the Affiliated Hospital of North Sichuan Medical College. Baseline information for the two groups is shown in Supplementary Table S1. The inclusion criteria for the PC group included (1) pathological diagnosis of PC; (2) complete follow-up information; and (3) absence of other malignant tumors prior to the operation and no previous antitumor therapy. Serum samples for the control group were collected from healthy individuals who underwent physical examinations and were matched by gender and age to the PC group. Blood samples were drawn into serum separator gel tubes without anticoagulants and centrifuged at 3500 rpm for 10 min. The resulting supernatant was transferred to 1.5 mL centrifuge tubes and stored at −80 °C for subsequent lipidomic analysis.

For lipid extraction, 20 μL of thawed serum was mixed with 120 μL of methanol and vortexed for 180 s. Then, 360 μL of methyl tert-butyl ether and 100 μL of water were added, followed by 10 min of vortexing. The mixture was centrifuged at 15,000× *g* for 15 min, after which 300 μL of the supernatant was carefully transferred to a fresh centrifuge tube. The sample was then dried in a vacuum concentrator. The dried lipid extracts were dissolved in a mixture of isopropanol, acetonitrile, and methanol (5:5:2) and quantified by adding the mixed internal standard. Samples for quality control (QC) were prepared by pooling the patient samples. The analysis of lipids was performed using a Shimadzu LC-20ADXR (Kyoto, Japan) coupled with a Sciex 4500MD triple quadrupole mass spectrometer (AB Sciex, Singapore). The lipids were chromatographically separated on an ACQUITY UPLC HSS C18 column (1.8 μm, 2.1 mm × 100 mm, Waters, Milford, MA, USA). The mobile phases were an acetonitrile–water solution (9:1) as phase A and an acetonitrile–water solution (5:5) as phase B, with 10 mM ammonium formate and 1% formic acid as additives in both phases. With an injection volume of 2 μL and a flow rate of 0.3 mL/min, the column temperature was kept at 50 °C. The detection mode was set at Selected Reaction Monitoring Mode, and the concentrations of lipids in each class were calculated via correction with isotope-labeled internal standards (SPLASH LIPIDOMIX, Avanti, Alexandria, VA, USA). Peak alignment and quantitative analysis were performed using Analyst MD software (version 1.7.3) and Multiquant MD (version 3.0.3). Finally, the resulting data were analyzed using a pseudo-targeted metabolomics approach and visualized using MetaboAnalyst 6.0 [33,34].

### 2.5. Identification of GLM-Related Molecular Subtypes in PC

A total of 930 PC samples were collected from the TCGA-PAAD, GSE57495 [35], GSE28735 [36,37], GSE62452 [38], E-MTAB-6134 [39], ICGC-CA, and ICGC-AU datasets for gene expression data and clinical annotation. Batch effects were mitigated using the “sva” package for correction [40].

The 930 PC samples were clustered by performing nonnegative matrix factorization (NMF) based on the expression of GMRGs [41]. The range of cluster counts, k, was from 2 to 10. The R package “NMF” was used to calculate the average profile width of the common membership matrix, which facilitated the classification of samples into distinct molecular subtypes. The prognostic efficacy of these clusters was evaluated through Kaplan–Meier analysis. Fifty hallmark pathways were downloaded from MsigDB and organized. Each pathway was scored for enrichment by single-sample gene set enrichment analysis (ssGSEA), and heatmaps were used to show differences in pathway enrichment scores between the 2 subtypes.

### 2.6. Analysis of TIME and Drug Sensitivity Based on GLM-Related Molecular Subtypes

The “Estimate” package was employed to predict the levels of stromal cells, immune cells, and tumor purity. Additionally, a series of methods, including TIMER, QUANTISEQ, MCPCOUNTER, XCELL, EPIC, and CIBERSORT, were employed to further analyze the TIME of different subtypes. Subsequently, differences in typical immune checkpoint genes (ICGs) between subtypes were explored, showing only statistically significant findings. In addition, the “oncoPredict” package was used to calculate the IC50 of commonly used chemotherapeutic drugs for different subtypes to guide personalized treatment for PC patients [42].

### 2.7. Identification of the Core GMRG in PC

Based on the RF algorithm, we ranked the GMRGs according to survival importance to identify the core GMRG in PC [43]. Meanwhile, we used the BEST website to further validate the prognostic value of the core GMRG across various PC datasets [44]. In addition, we further investigated the relationship between the core gene and clinicopathologic features of PC.

### 2.8. Real-Time Quantitative PCR of PC Cell Lines and Tissue Samples

The expression levels of ALDH2 were investigated using three PC cell lines (SW-1990, Mia-paca-2, and Panc-1) and a normal pancreatic cell line (H6C7). All four cell lines were cultured in DMEM supplemented with 10% fetal bovine serum and a penicillin–streptomycin solution (penicillin: 100 U/mL; streptomycin: 0.1 mg/mL). A 37 °C cell culture incubator with 5% CO_2_ was used to keep the cells alive. In addition, we obtained cancerous and peritumoral tissues from seven PC patients who underwent surgery at the First Affiliated Hospital of Dalian Medical University between 2023.01 and 2023.12. All samples were stored at −80 degrees Celsius before RNA extraction. The conventional TRIzol procedure was used to extract total RNA from the four cell lines and tissue samples, and a reverse transcription kit was used to create cDNA. A fluorescent dye-based assay based on SYBR Green I was then used to evaluate the target genes’ expression levels. RNA levels were analyzed and quantified using the 2^−∆∆Ct^ method. The primers are as follows: β-actin, (forward) CCTGGGCATGGAGTCCTGTG and (reverse) TCTTCATTGTGCTGGGTGCC; ALDH2, (forward) GAAGATCCTCGGCTACATCAACAC and (reverse) GTAACCACGGTCAGCAGCAATG.

### 2.9. Immunohistochemical Analysis of ALDH2 Expression and Its Correlation with Clinicopathologic Features in PC

The paraffin-embedded pathological sections of 62 PC tissues from patients who underwent surgery between January 2017 and June 2023, along with peritumoral tissues from 10 of these patients, were collected from the First Hospital of Dalian Medical University. Clinical information for the PC samples is shown in Supplementary Table S2. Primary antibodies (Proteintech 15310-1-AP, 1:200, Rosemont, IL, USA) were incubated with the tissue sections at 4 °C overnight. The sections were then treated with the corresponding secondary antibodies for 50 min at room temperature. Finally, the immunohistochemical images were quantitatively analyzed using ImageJ software (version 1.54). Positive signals were quantified by calculating the mean optical density values. The clinicopathological characteristics of the patients were obtained from our hospital’s system, and survival information was collected through telephone follow-up until February 2025.

## 3. Results

### 3.1. Pan-Cancer Characterization of GMRGs

**Figure 1:**
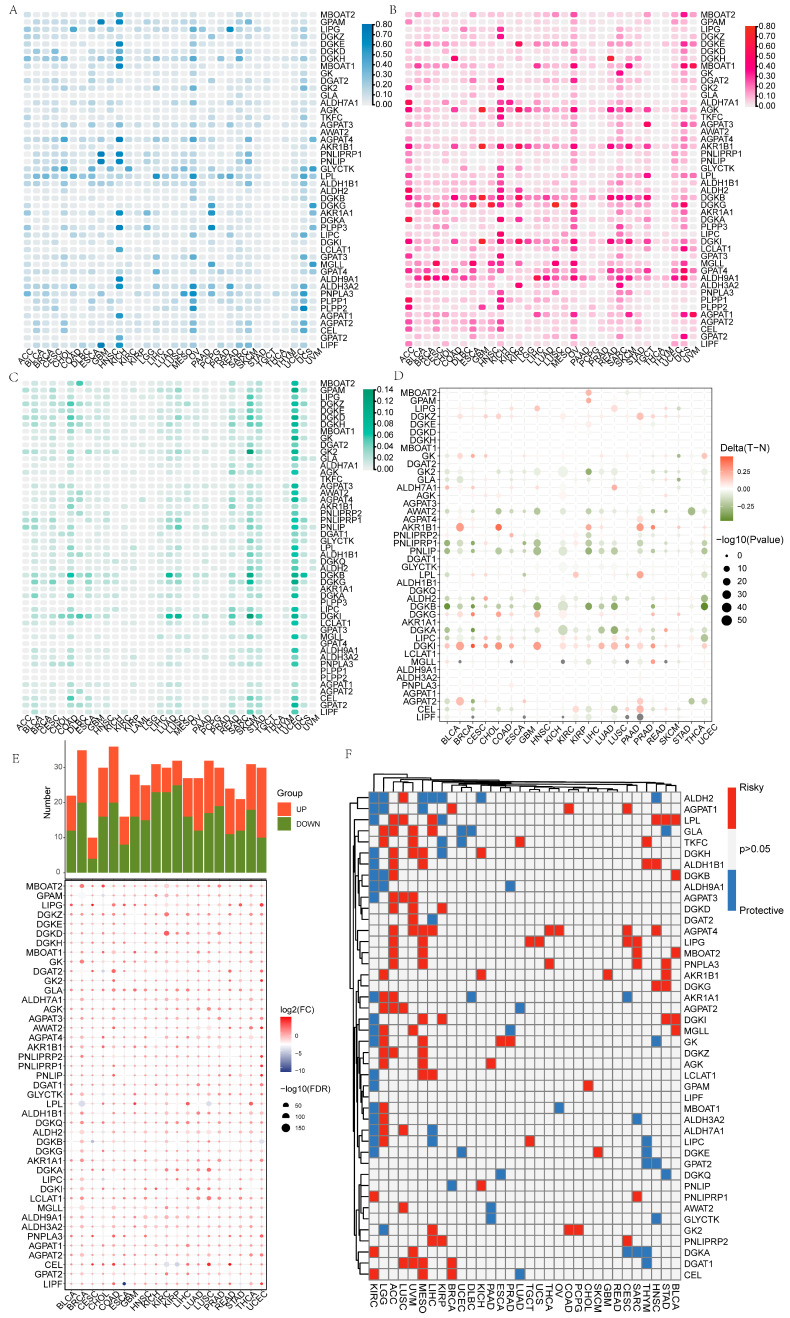
Pan-cancer overview of GMRGs. (**A**) GMRGs’ CNV deletion features in different cancers. (**B**) GMRGs’ CNV amplification features in different cancers. (**C**) GMRGs’ SNV features in different cancers. (**D**) GMRGs’ methylation features in different cancers. (**E**) GMRGs’ expression features in different cancers. (**F**) GMRGs’ prognostic significance in different cancers. Protective genes: HR < 1 and *p* < 0.05; risk genes: HR > 1 and *p* < 0.05.

The role of GMRGs in the development of human malignant tumors has not been elucidated, so we first summarized the pan-cancer characteristics of GMRGs using data from TCGA. We found that more GMRGs, including aldehyde dehydrogenase 3 family member A2 (ALDH3A2), glycerate kinase (GLYCTK), lipoprotein lipase (LPL), and diacylglycerol kinase eta (DGKH), exhibited more CNV deletions in kidney chromophobe (KICH), ovarian serous cystadenocarcinoma (OV), and uterine carcinosarcoma (UCS) (Figure 1A). More GMRGs, including diacylglycerol kinase beta (DGKB), diacylglycerol kinase iota (DGKI), acylglycerol kinase (AGK), aldo-keto reductase family 1 member B1 (AKR1B1), and aldehyde dehydrogenase 9A1 (ALDH9A1), exhibited more CNV amplifications in adrenocortical carcinoma (ACC), KICH, OV, and UCS (Figure 1B). In addition, skin melanoma (SKCM) and endometrial cancer (UCEC) had a high number of GMRGs with SNVs, specifically 38 GMRGs in SKCM and 40 GMRGs in UCEC (Figure 1C). Considering that aberrant DNA methylation is also closely associated with tumorigenesis [45], we similarly analyzed the DNA methylation of GMRGs in different tumor types. Figure 1D shows that pancreatic lipase-related protein 1 (PNLIPRP1), pancreatic lipase (PNLIP), and DGKB presented hypomethylation in bladder urothelial carcinoma (BLCA), head and neck squamous cell carcinoma (HNSC), liver hepatocellular carcinoma (LIHC), lung squamous cell carcinoma (LUSC), and uterine corpus endometrial carcinoma (UCEC), whereas DGKI and AKR1B1 presented hypermethylation in breast invasive carcinoma (BRCA), colon adenocarcinoma (COAD), and prostate adenocarcinoma (PRAD) (−log_10_Pvalue ≤ 10). In addition, the expression levels of most GMRGs were different compared with those in control samples, and more GMRGs were downregulated in tumors (Figure 1E). Then, the prognostic value of GMRGs in different tumor types was investigated in depth by analyzing the gene expression levels and the survival time. It was found that most GMRGs, such as ALDH2, LPL, and DGKH, acted as protective genes (HR < 1 and *p* < 0.05) in renal clear cell carcinoma (KIRC), while most GMRGs acted as risk genes (HR > 1 and *p* < 0.05) in ACC, LUSC, uveal melanoma (UVM), and mesothelioma (MESO) (Figure 1F).

### 3.2. scRNA-seq Data Revealed the Presence of Aberrant GLM in PC

**Figure 2:**
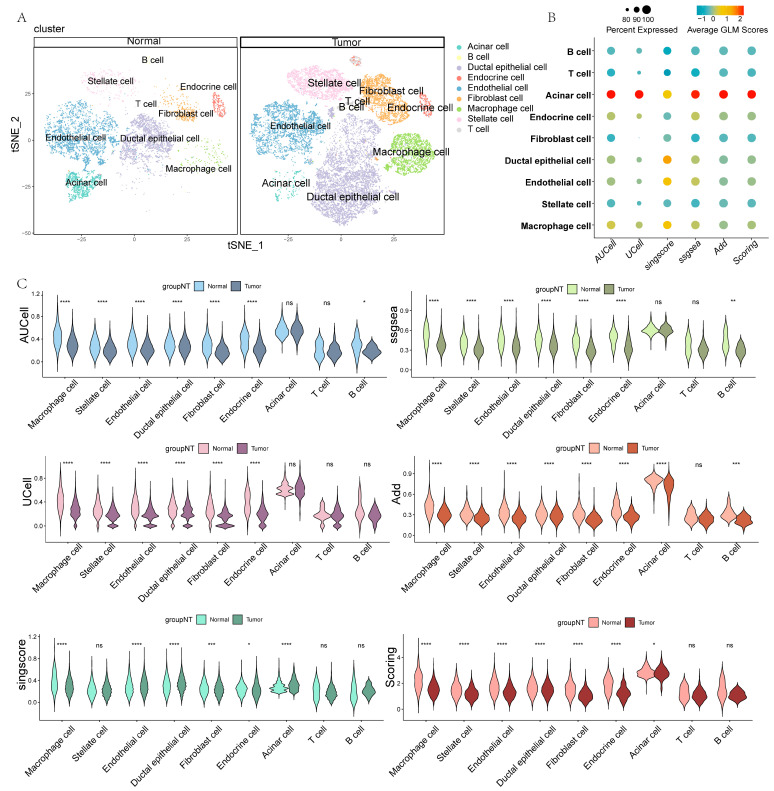
GLM characterization of PC based on scRNA-seq. (**A**) Annotation results for different clustered cells. (**B**) The bubble plot shows the GLM scores for different cell types. (**C**) The violin charts show the GLM scores based on different methods (ns, non-significant, * *p* < 0.05, ** *p* < 0.01, *** *p* < 0.001, and **** *p* < 0.0001).

After the cell annotation, the cell clusters were classified into nine cell types: acinar cells, B cells, ductal epithelial cells, endocrine cells, endothelial cells, fibroblasts, macrophages, stellate cells, and T cells (Supplementary Figure S1A and Figure 2A). To explore GLM characteristics in PC tissues, six methods were utilized to score GLM signals in scRNA-seq data. Acinar cells showed significantly active GLM signaling, whereas the opposite was true for T cells (Figure 2B). Importantly, the different cell types within tumor tissues all exhibited lower GLM signals compared with normal tissues, indicating a potential role of GLM in PC (Figure 2C). To more clearly visualize the GLM signal of each cell, we further projected the GLM scores onto the previously generated t-Stochastic Neighbor Embedding (t-SNE) plot (Supplementary Figure S1B).

In addition, we categorized ductal cells from PC tissues into aneuploid and diploid cells using “CopyKat” and illustrated their distribution in a t-SNE plot (Supplementary Figure S2A). Subsequently, we further compared the differences in GLM scores between aneuploid and diploid cells. The results showed that aneuploid cells exhibited higher GLM scores (Supplementary Figure S2B,C). The ductal cells used for benign and malignant identification were all derived from tumor tissue, so this difference may be related to gene dosage effects caused by cellular aneuploidy or adaptive reprogramming of signaling pathways. Finally, we generated spatially resolved imaging maps of three cases of PC using spatial transcriptomic techniques, revealing the spatial distribution of GLM scores in PC tissues for the first time (Supplementary Figure S3).

### 3.3. Characterization of GLM in PC Based on Lipidomic Analysis

**Figure 3:**
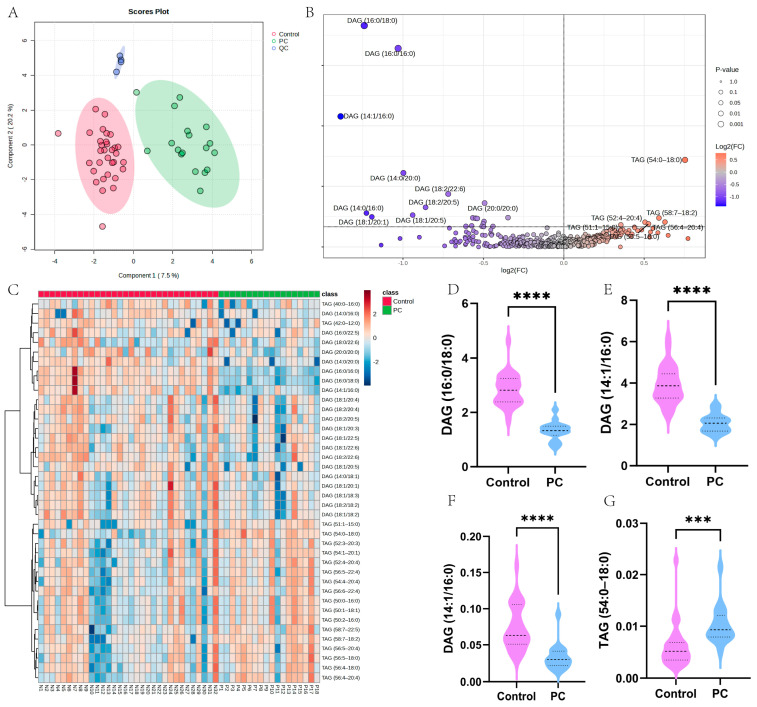
Characterization of the GLM landscape of PC based on lipidomic analysis. (**A**) sPLS-DA analysis of the overall sample. Green dots represent PC samples, red dots represent control samples, and blue dots represent QC samples. (**B**) Differential analysis of glycerolipid-related metabolites between PC and control samples. (**C**) A heatmap of glycerolipid-related metabolites. (**D**–**G**) The expression levels of four significantly different metabolites (*** *p* < 0.001; **** *p* < 0.0001).

To further validate the aberrant GLM profile in PC patients, we performed serum lipidomic analysis. In total, 50 serum samples were included in this study, including 18 samples from pathologically diagnosed PC and 32 samples from healthy control subjects matched to the gender and age of the PC patients. sPLS-DA analysis showed the aggregation of QC samples and significant separation between the two groups (Figure 3A). To further characterize the GLM profiles of PC patients, we performed a differential analysis of GLM-related metabolites, including TAG and DAG. We found that TAG-related metabolites were significantly upregulated in PC, while DAG-related metabolites were significantly downregulated (Figure 3B). In addition, we further plotted heatmaps to visualize the top 40 differential metabolites. TAG-related metabolites were all upregulated in PC, while the opposite was true for DAG (Figure 3C). In addition, the four metabolites with significant differences were visualized using box plots (Figure 3D–G). The lipidomic results further demonstrated that GLM was significantly dysregulated in PC patients.

### 3.4. Development of GLM-Related Molecular Subtypes Based on Bulk RNA-seq

**Figure 4:**
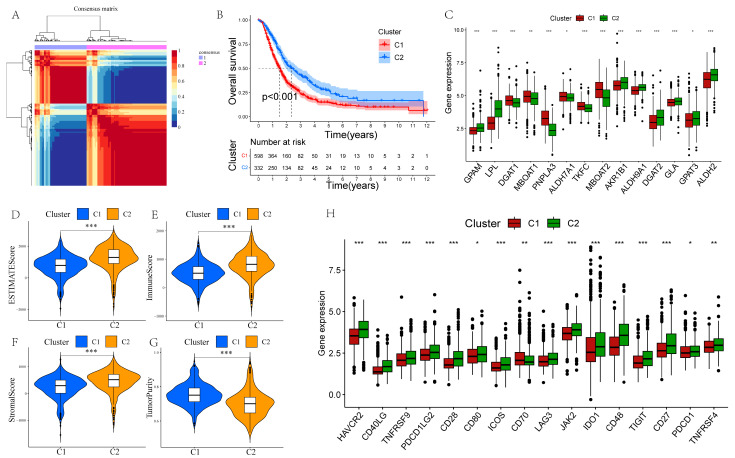
NMF clustering identification of two molecular subtypes in PC. (**A**) The ideal number for clustering is 2. (**B**) Prognostic analysis of the two subtypes. (**C**) Differential expression analysis of GMRGs. (**D**–**G**) Estimatescore, immunescore, stromalscore, and tumor purity are compared between the two subtypes. (**H**) The differences between the two subtypes in terms of ICG expression (* *p* < 0.05, ** *p* < 0.01, and *** *p* < 0.001).

The scRNA-seq and lipidomic data showed the presence of significantly dysregulated GLM in PCs, suggesting an important role of GLM in PC, so we further investigated the GLM-related molecular subtypes of PC. Based on the expression levels of GMRGs, 930 PC samples were categorized into two subtypes (C1 and C2; Figure 4A), and prognostic analyses demonstrated that the C2 subtype was associated with a significantly better prognosis than C1 (Figure 4B). In addition, Figure 4C demonstrates significantly different expression levels of GMRGs in different subtypes. To fully explore the intrinsic molecular characteristics of different subtypes of PC patients, we assessed the differences in the enrichment of hallmarks in different subtypes. The heatmap shows that a majority of these pathways were statistically different between the two subtypes (Supplementary Figure S4A). Specifically, cell-proliferation- and cycle-regulation-related pathways, including mitotic spindle, G2M checkpoints, and E2F targets, were enriched in the C1 subtype, and many metabolism-related pathways, such as bile acid metabolism, exogenous metabolism, and fatty acid metabolism, were significantly enriched in C2.

### 3.5. Differences in TIME Between Different Subtypes

Subsequently, we calculated the immunescore, stromalscore, estimatescore, and tumor purity for the two subtypes. We found that C1 had higher tumor purity with lower proportions of stromal cells and immune cells (Figure 4D–G). To further investigate the differences in TIME between C1 and C2, we analyzed immune cell infiltration using seven methods. The results indicated that immune cell infiltration was significantly higher in the C2 subtype compared to the C1 subtype, particularly for M2 macrophages, CD8+ and CD4+ T cells, and endothelial cells (Supplementary Figure S4B). Additionally, all ICGs except CD70 were significantly upregulated in the C2 subtype (Figure 4H).

### 3.6. GLM-Related Molecular Subtypes Can Guide Personalized Therapy for PC

**Figure 5:**
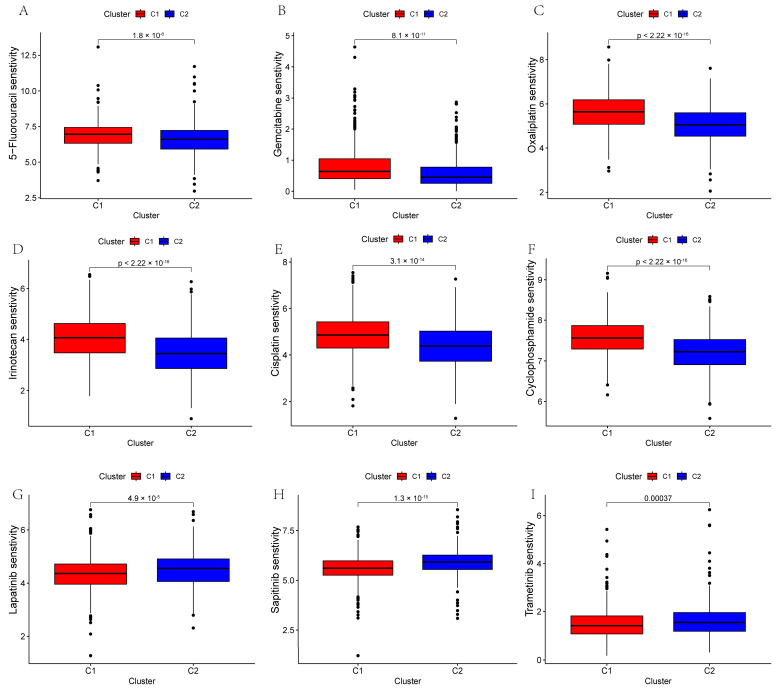
The plots show the estimated IC50 of chemotherapeutic drugs for the two subtypes. (**A**–**I**) 5-Fluorouracil, gemcitabine, oxaliplatin, irinotecan, cisplatin, cyclophosphamide, lapatinib, sapitinib, and trametinib.

To investigate the potential value of GLM-related molecular subtypes in drug treatments for PC patients, we predicted the therapeutic responses of 930 PC patients to different drugs. The results show that patients with the C2 subtype showed higher sensitivity to 5-fluorouracil, gemcitabine, oxaliplatin, irinotecan, cisplatin, and cyclophosphamide, while patients with the C1 subtype showed higher sensitivity to lapatinib, sapitinib, and tetracycline (Figure 5A–I).

### 3.7. Identification and Validation of the Prognostic Target in PC

**Figure 6:**
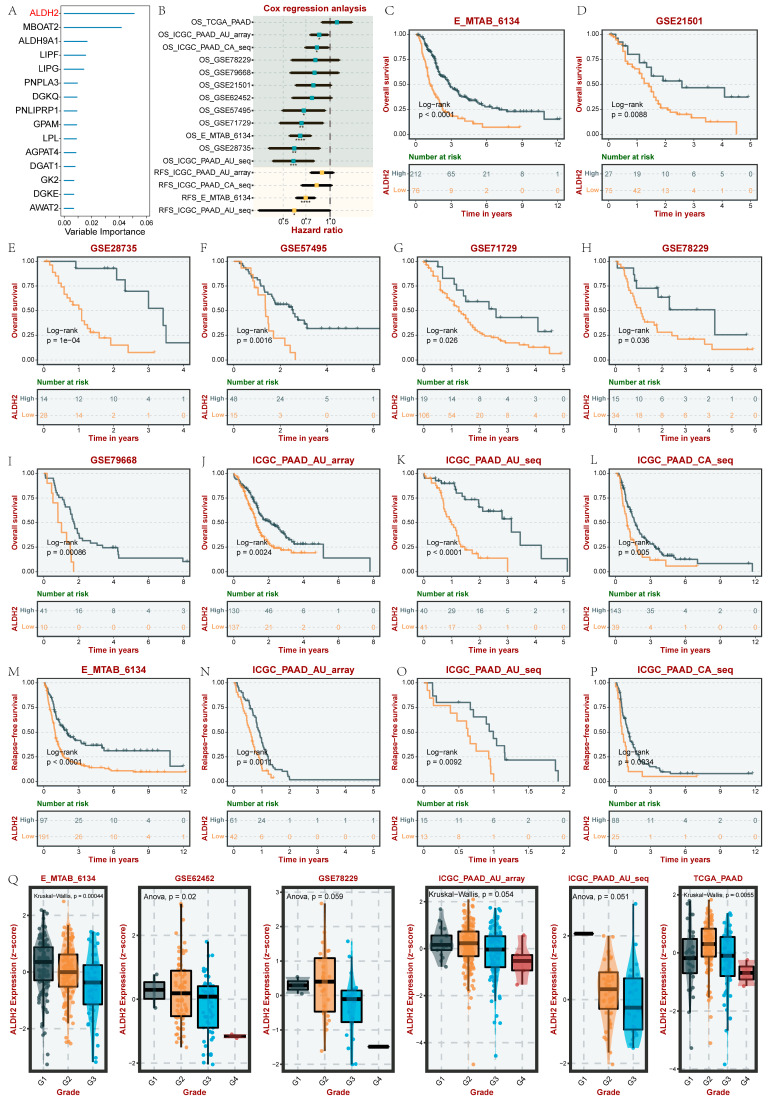
The identification of the key prognostic GMRG in PC. (**A**) RF-based identification of key GMRGs. (**B**) Cox regression analysis of ALDH2 in several PC datasets. (**C**–**P**) Kaplan–Meier analyses (OS and RFS) of ALDH2 in several PC datasets. (**Q**) Analysis of the correlation between ALDH2 and the PC tumor grade. * *p* < 0.05, ** *p* < 0.01, *** *p* < 0.001, and **** *p* < 0.0001.

To further identify the core GMRG in PC, we used the RF algorithm to rank the GMRGs based on their prognostic significance in 930 PC samples. We found that ALDH2 had the most significant importance (importance score > 0.05) in influencing the prognosis of PC (Figure 6A). Subsequently, we further explored the prognostic significance of ALDH2 in various datasets of PC using the BEST database. Figure 6B demonstrates the Cox regression analysis results, including overall survival (OS) and recurrence-free survival (RFS), which revealed that ALDH2 was a protective factor in PC prognosis in nine datasets (HR < 1, *p* < 0.05). The Kaplan–Meier curve was then plotted, and the results of multiple datasets further revealed that ALDH2 was closely associated with a better prognosis for PC (Figure 6C–P). In addition, we also found that ALDH2 was closely associated with a lower tissue grade in PC (Figure 6Q).

### 3.8. The Expression of ALDH2 and Its Association with Clinicopathological Parameters in PC

**Figure 7:**
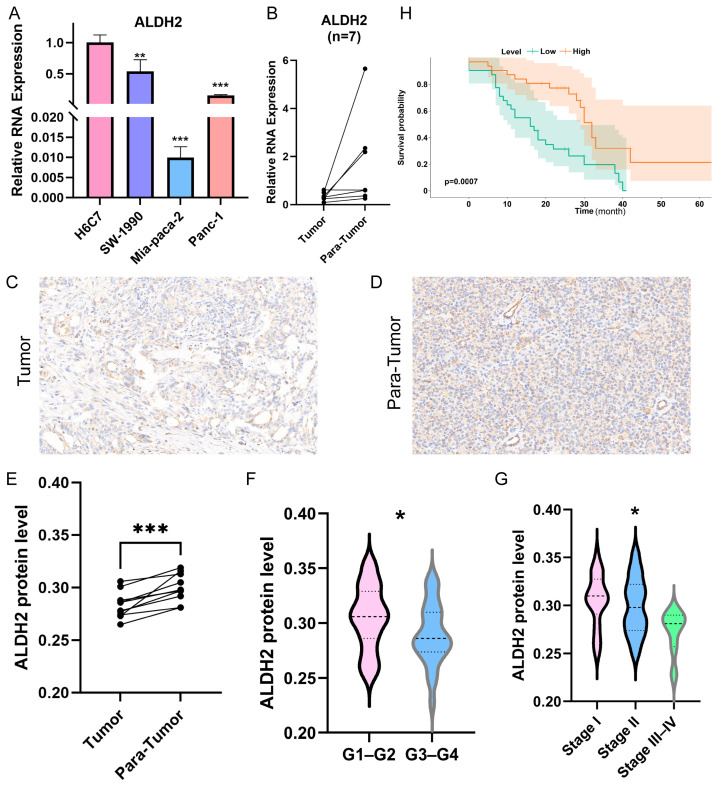
Cell lines and clinical cohort validation of ALDH2. (**A**) The mRNA expression levels of ALDH2 in H6C7 and three PC cell lines. (**B**) The mRNA expression levels of ALDH2 in clinical tissues. (**C**,**D**) Immunohistochemical images of tumor and para-tumor tissues. (**E**) The protein levels of ALDH2 in 10 pairs of PC and para-tumor tissues. (**F**) The protein levels of ALDH2 between pathological grade 1–2 and pathological grade 3–4 PC tissues. (**G**) The protein levels of ALDH2 among stage I, stage II, and stage III–IV PC tissues. (**H**) Survival curves of PC patients in the high- and low-ALDH2-expression groups (* *p* < 0.05, ** *p* < 0.01, and *** *p* < 0.001).

We then further explored the expression profile of ALDH2 in PC by performing PCR. The RNA expression levels of ALDH2 in different PC cell lines (SW-1990, Mia-paca-2, and Panc-1) and the human normal pancreatic cell line H6C7 were first compared. We found that the RNA expression of ALDH2 was significantly downregulated in three PC cell lines (Figure 7A). We also compared the RNA expression of ALDH2 in cancerous and peritumoral tissues from seven PC patients who underwent surgery, and the results showed that the RNA expression of ALDH2 was lower in cancer tissues (Figure 7B).

To further validate the significance of ALDH2 in PC, we performed immunohistochemistry to assess its protein levels in both cancerous and paraneoplastic tissues. The results revealed that ALDH2 expression was significantly downregulated in PC tissues (Figure 7C–E). In addition, Figure 7F–G reveal that ALDH2 expression levels were lower in cases with higher tumor grades (*t* test, *p* < 0.05) and later pathological stages (Kruskal–Wallis test, *p* < 0.05). Finally, based on the median expression level of ALDH2, we analyzed its association with prognosis in 62 PC patients (Figure 7H). The findings indicated that reduced ALDH2 levels were associated with a poor prognosis.

## 4. Discussion

PC is a highly lethal malignancy of the gastrointestinal tract and is known for its early metastasis and resistance to anticancer therapies [46]. Improvements in therapeutic approaches over the past few years have improved the treatment and prognosis of PC, but survival rates remain low. In recent years, there has been growing evidence that lipid metabolism can promote tumorigenesis and disease progression and contribute to drug resistance [47]. As an important class of lipids, glycerolipids play key roles in human physiology and disease. Glycerolipid-metabolizing enzymes regulate these metabolic signaling pathways, which have become attractive targets for cancer therapeutic interventions [16]. Therefore, a complete understanding of reprogrammed GLM is key to identifying new biomarkers and providing novel insights for targeting metabolic networks in PC.

Given the important role of GLM in malignant tumors, in this study, we first prepared a comprehensive summary of the transcriptomic profiles of GMRGs in different types of malignant tumors. CNVs and SNVs are thought to have significant effects on cellular disorders that may lead to cancer. ALDH3A2, GLYCTK, LPL, and DGKH showed a high frequency of CNV deletions in KICH, OV, and UCS, whereas DGKB, DGKI, AGK, AKR1B1, and ALDH9A1 tended more toward CNV amplifications. The imbalance of CNVs in these genes may drive tumor development by interfering with cellular metabolic homeostasis, oxidative stress regulation, and signaling pathway activation. The co-existence of GMRG deletion and amplification in KICH, OV, and UCS suggests that their metabolic plasticity may be dependent on “dual regulation”. SKCM, a cancer type with one of the highest mutation loads, is enriched in GMRG SNVs, which may reflect a balancing mechanism between oxidative stress tolerance and metabolic adaptations. The high frequency of SNVs in GMRGs in UCEC may be associated with estrogen metabolism and an inflammatory microenvironment. DNA methylation is intimately linked to genomic regulation, and defects in this process could lead to tumor suppressor-gene silencing and cell-cycle and DNA-repair dysfunction. Notably, we found altered CNVs, SNVs, and methylation of GMRGs in various malignant tumors, which may contribute to tumor development, necessitating further detailed studies of GMRGs.

Subsequently, to further explore the potential role of GLM in PC, we analyzed GMRGs at the scRNA-seq level. The scRNA-seq data revealed that the GLM enrichment score was highest in acinar cells in both normal and PC samples. More importantly, there was a significant difference in GLM scores between PC and normal samples of either cell type. Specifically, the GLM signaling pathway was more inactive in PC samples compared to normal samples, suggesting that the GLM pathway may have a potential anticancer role. To validate the aforementioned discoveries at more levels, we performed a lipidomic analysis on 50 clinical serum samples. The results showed significant abnormalities in GLM in PC: specifically, TAG-related metabolites were significantly upregulated and DAG-related metabolites were significantly downregulated in PC, suggesting that GLM reprogramming plays an important role in PC.

In recent years, molecular classification has been considered a promising tool to optimize prognostic prediction and precision medicine in cancer patients. It helps to classify patients into groups that are less heterogeneous and more appropriate in terms of therapeutic response, leading to better predictions of disease evolution and better personalization of treatment. Given the important role of GLM in PC, we identified two GLM-related molecular subtypes in 930 PC samples. Prognostic analyses revealed significant differences in prognosis between the two subtypes. In an enrichment analysis of 50 hallmark pathways, the C1 subtype was found to be predominantly enriched in cell proliferation-related pathways, such as mitotic spindle and G2M checkpoint, whereas the C2 subtype was predominantly enriched in metabolism-related pathways, such as fatty acid metabolism and bile acid metabolism. The significant differences in biological functions between the different subtypes may be one of the main reasons for their prognostic differences.

The complex TIME of PC is a key determinant of the prognosis and drug sensitivity of PC patients, and a comprehensive characterization of the TIME of different subtypes is necessary to advance the precision treatment of PC [48]. We found that the C2 subtype, with a better prognosis, had a greater abundance of immune cells. This finding has important implications because differences in TIME between subtypes may partially explain the poorer prognosis of the C1 subtype. In addition, the C2 subtype commonly had higher expression levels of ICGs, and the higher abundance of immune cells in this subtype may be a compensatory phenomenon due to high levels of ICGs. More importantly, the C2 subtype is more sensitive to clinically used chemotherapeutic agents, such as 5-fluorouracil and gemcitabine. This suggests that GLM-based molecular subtypes can guide personalized clinical dosing, minimize the risk of toxicity from chemotherapy, and provide a long-term survival benefit.

scRNA-seq and lipidomic analysis revealed the important role of GLM reprogramming in PC, and the establishment of bulk-RNA-based GLM-related molecular subtypes further demonstrated the potential of GLM in the prognosis and treatment of PC. Therefore, we further searched for the most central GMRGs affecting the prognosis of PC based on the RF algorithm. ALDH2 is a mitochondrial enzyme that plays a key role in the removal of endogenous aldehydes produced by lipid peroxidation triggered by oxidative stress. A series of basic studies and clinical trials have shown that ALDH2 may affect lipid metabolism. For example, a deficiency of ALDH2 caused severe dyslipidemia in mice [49]. The knock-in of the ALDH2 rs671 A allele significantly increased plasma and liver cholesterol levels [50]. However, the overexpression of ALDH2 reduces lipid deposition [51]. A meta-analysis showed that genetic variation in ALDH2 was associated with triglyceride levels and could be used as a genetic marker for lipid dyslipidemia in East Asian populations [52]. In addition, the expression of ALDH2 has been found to be a potential prognostic indicator for a number of malignancies; e.g., the downregulation of ALDH2 expression was associated with poorer prognosis in hepatocellular carcinoma [53], melanoma [54], and lung adenocarcinoma [55]. However, the role of ALDH2 expression in PC has not been reported. We hypothesize that ALDH2 may inhibit PC progression by regulating lipid metabolism. Our study is the first to demonstrate that ALDH2 expression is downregulated in PC, while high ALDH2 expression is associated with an earlier stage, a lower pathologic grade, and a better prognosis in PC patients. This has been confirmed not only in comprehensive public database analyses, but also in a large number of clinical tissue samples.

To our knowledge, this is the first multi-omics study to explore the potential role of GLM in PC. In addition, we recognize some limitations of the present study: (1) Although aberrant GLM in PC was demonstrated based on multi-omics analyses, these data do not provide a comprehensive picture of the dynamic process of GLM. (2) The characterization of GLM in PC offers valuable insights into potential therapeutic regimens. However, such data must undergo rigorous prospective validation before being implemented in clinical practice. (3) Only the expression and prognostic characterization of ALDH2 in PC were validated; further mechanistic experiments are still needed to explore the mechanism of ALDH2’s action in PC.

## 5. Conclusions

In conclusion, we demonstrated that GLM reprogramming occurs in PC by performing a multi-omics analysis and described two GLM-related subtypes with different biological characteristics, prognoses, and therapeutic responses. Meanwhile, we demonstrated that ALDH2 might serve as a new tumor marker and provide a basis for inhibiting PC progression.

## Data Availability

The datasets analyzed in the current study are available in TCGA (https://portal.gdc.cancer.gov/ (accessed on 15 May 2022)), GEO (https://www.ncbi.nlm.nih.gov/geo/query/acc.cgi?acc=GSE57495, https://www.ncbi.nlm.nih.gov/geo/query/acc.cgi?acc=GSE28735, https://www.ncbi.nlm.nih.gov/geo/query/acc.cgi?acc=GSE62452 (accessed on 15 May 2022)), ICGC (https://dcc.icgc.org), and ArrayExpress (https://www.ebi.ac.uk/biostudies/arrayexpress/studies/E-MTAB-6134 (accessed on 15 May 2022)). The lipidomic data are not publicly available due to privacy. Further inquiries can be directed to the corresponding authors.

## Supplementary Materials

The following supporting information can be downloaded at https://www.mdpi.com/article/10.3390/metabo15030207/s1, Figure S1: scRNA-seq data annotation and visualization of GLM scores. (A) Cell-type annotation. (B) The single-cell distribution of GLM scores is shown using t-SNE; Figure S2: CopyKat analysis of scRNA-seq data. (A) The CopyKat findings are shown using t-SNE. (B–C) The differences in GLM scores between aneuploid and diploid (**** *p* < 0.0001); Figure S3: Spatial transcriptomics characteristics of GLM scores in three PC samples; Figure S4: Differences in biological function and immune cell infiltration between two subtypes. (A) The differences in hallmarks between the two subtypes are shown in the heatmap. (B) The differences between the two subtypes in terms of immunocyte infiltration (* *p* < 0.05, ** *p* < 0.01, and *** *p* < 0.001); Table S1: Clinical information for samples from the Affiliated Hospital of North Sichuan Medical College; Table S2: Clinical information for patients with pancreatic cancer from the First Affiliated Hospital of Dalian Medical University.

## Abbreviation

ACC	Adrenocortical carcinoma
BLCA	Bladder urothelial carcinoma
BRCA	Breast invasive carcinoma
CESC	Cervical squamous cell carcinoma and endocervical adenocarcinoma
CHOL	Cholangiocarcinoma
COAD	Colon adenocarcinoma
DLBC	Lymphoid neoplasm diffuse large B-cell lymphoma
ESCA	Esophageal carcinoma
GBM	Glioblastoma multiforme
HNSC	Head and neck squamous cell carcinoma
KICH	Kidney chromophobe
KIRC	Kidney renal clear cell carcinoma
KIRP	Kidney renal papillary cell carcinoma
LGG	Brain lower-grade glioma
LIHC	Liver hepatocellular carcinoma
LUAD	Lung adenocarcinoma
LUSC	Lung squamous cell carcinoma
MESO	Mesothelioma
OV	Ovarian serous cystadenocarcinoma
PAAD	Pancreatic adenocarcinoma
PCPG	Pheochromocytoma and paraganglioma
PRAD	Prostate adenocarcinoma
READ	Rectum adenocarcinoma
SARC	Sarcoma
SKCM	Skin cutaneous melanoma
STAD	Stomach adenocarcinoma
TGCT	Testicular germ cell tumors
THYM	Thymoma
THCA	Thyroid carcinoma
UCS	Uterine carcinosarcoma
UCEC	Uterine corpus endometrial carcinoma
UVM	Uveal melanoma
GLM	Glycerolipid metabolism
PC	Pancreatic cancer
GMRG	Glycerolipid metabolism-related gene
CNV	Copy number variants
SNV	Single nucleotide variations
scRNA-seq	Single-cell RNA sequencing
TAG	Triacylglycerol
DAG	Diacylglycerol
MAG	Monoacylglycerol
TIME	Tumor immune microenvironment
RF	Random forest
QC	Quality control
NMF	Nonnegative matrix factorization
ssGSEA	Single sample gene set enrichment analysis
ICG	Immune checkpoint gene
t-SNE	t-Stochastic Neighbor Embedding
OS	Overall survival
RFS	Recurrence-free survival
ALDH2	Aldehyde dehydrogenase 2
ALDH3A2	Aldehyde dehydrogenase 3 family member A2
ALDH9A1	Aldehyde dehydrogenase 9A1
DGKB	Diacylglycerol kinase beta
DGKH	Diacylglycerol kinase eta
DGKI	Diacylglycerol kinase iota
AGK	Acylglycerol kinase
AKR1B1	Aldo-keto reductase family 1 member B1
GLYCTK	Glycerate kinase
LPL	Lipoprotein lipase
