# Supplementary material for: Multi-Omics Profiling Reveals Glycerolipid Metabolism-Associated Molecular Subtypes and Identifies ALDH2 as a Prognostic Biomarker in Pancreatic Cancer
Source: Metabolites. 2025 Mar 18;15(3):207. doi: 10.3390/metabo15030207 (PMC11943634; doi:10.3390/metabo15030207)
Supplement: Supplementary file 1 [file metabolites-15-00207-s001.zip › Table S1.pdf]

Table S1. Clinical information for samples from the Affiliated Hospital of North Sichuan Medical College.

	PC (N=18)	Control (N=32)	Statistic	P-value
**Gender**				
Female	3 (16.7%)	10 (31.3%)	$\chi^2 = 0.628$	0.428
Male	15 (83.3%)	22 (68.8%)		
**Age**				
Mean (SD)	64.6 (9.52)	64.8 (3.84)	W = 292	0.943
