# Supplementary material for: Multi-Omics Profiling Reveals Glycerolipid Metabolism-Associated Molecular Subtypes and Identifies ALDH2 as a Prognostic Biomarker in Pancreatic Cancer
Source: Metabolites. 2025 Mar 18;15(3):207. doi: 10.3390/metabo15030207 (PMC11943634; doi:10.3390/metabo15030207)
Supplement: Supplementary file 1 [file metabolites-15-00207-s001.zip › Table S2.pdf]

Table S2. Clinical information for patients with pancreatic cancer from the First Affiliated Hospital of Dalian Medical University.

Characteristics		Number	Percentage (%)
Age	<=65	34	54.8
	>65	28	45.2
Gender	Male	39	62.9
	Female	23	37.1
Clinical stage	Stage I	25	40.3
	Stage II	30	48.4
	Stage III-IV	7	11.3
Pathological grade	G1-2	35	56.5
	G3-4	25	40.3
	Unrecorded	2	3.2
