# Supplementary figures and images for: Multi-Omics Profiling Reveals Glycerolipid Metabolism-Associated Molecular Subtypes and Identifies ALDH2 as a Prognostic Biomarker in Pancreatic Cancer
Source: Metabolites. 2025 Mar 18;15(3):207. doi: 10.3390/metabo15030207 (PMC11943634; doi:10.3390/metabo15030207)

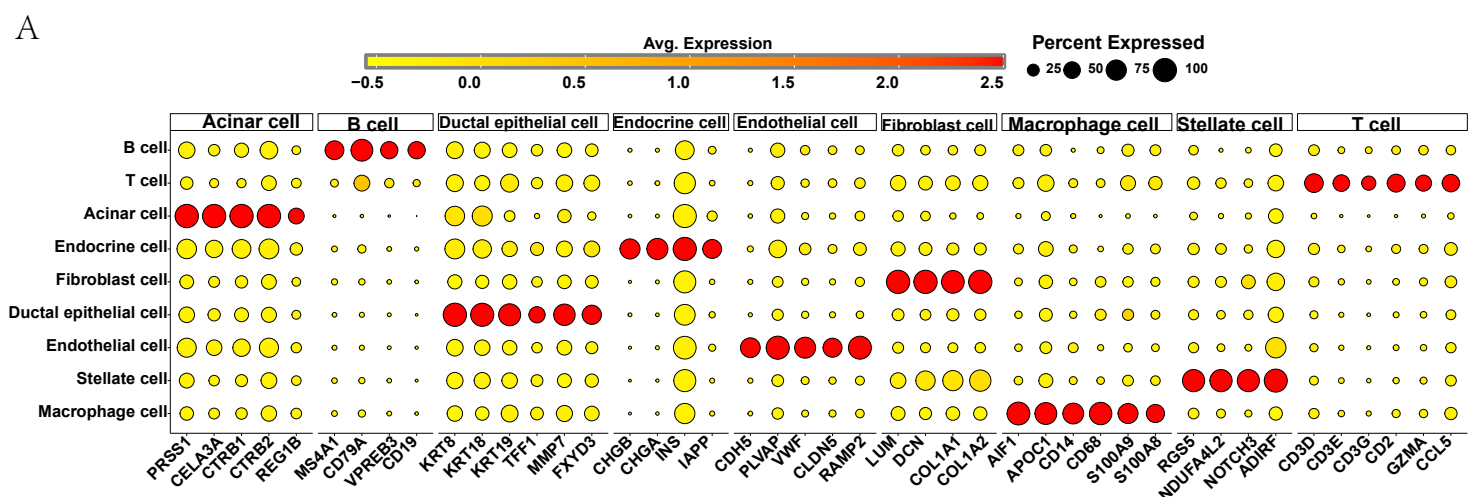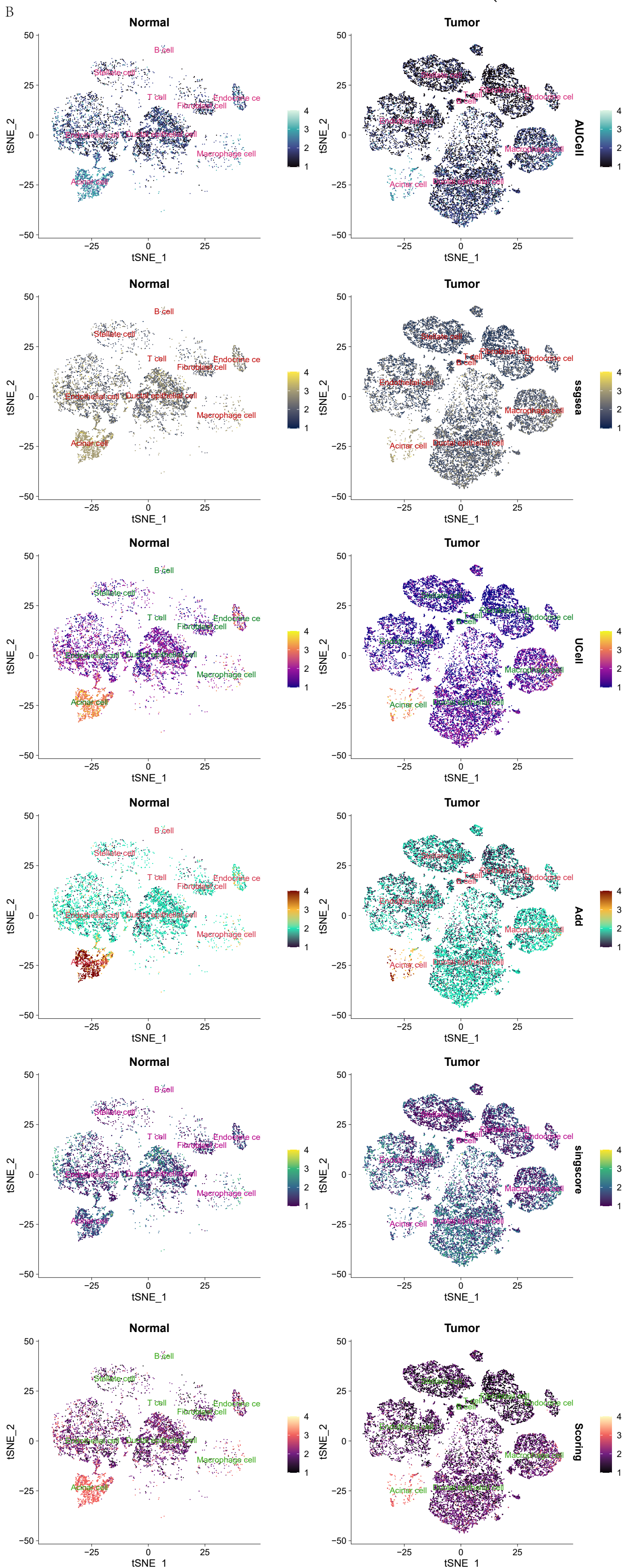

Supplement: Supplementary file 1 [file metabolites-15-00207-s001.zip › Supplementary Figure S1.pdf]

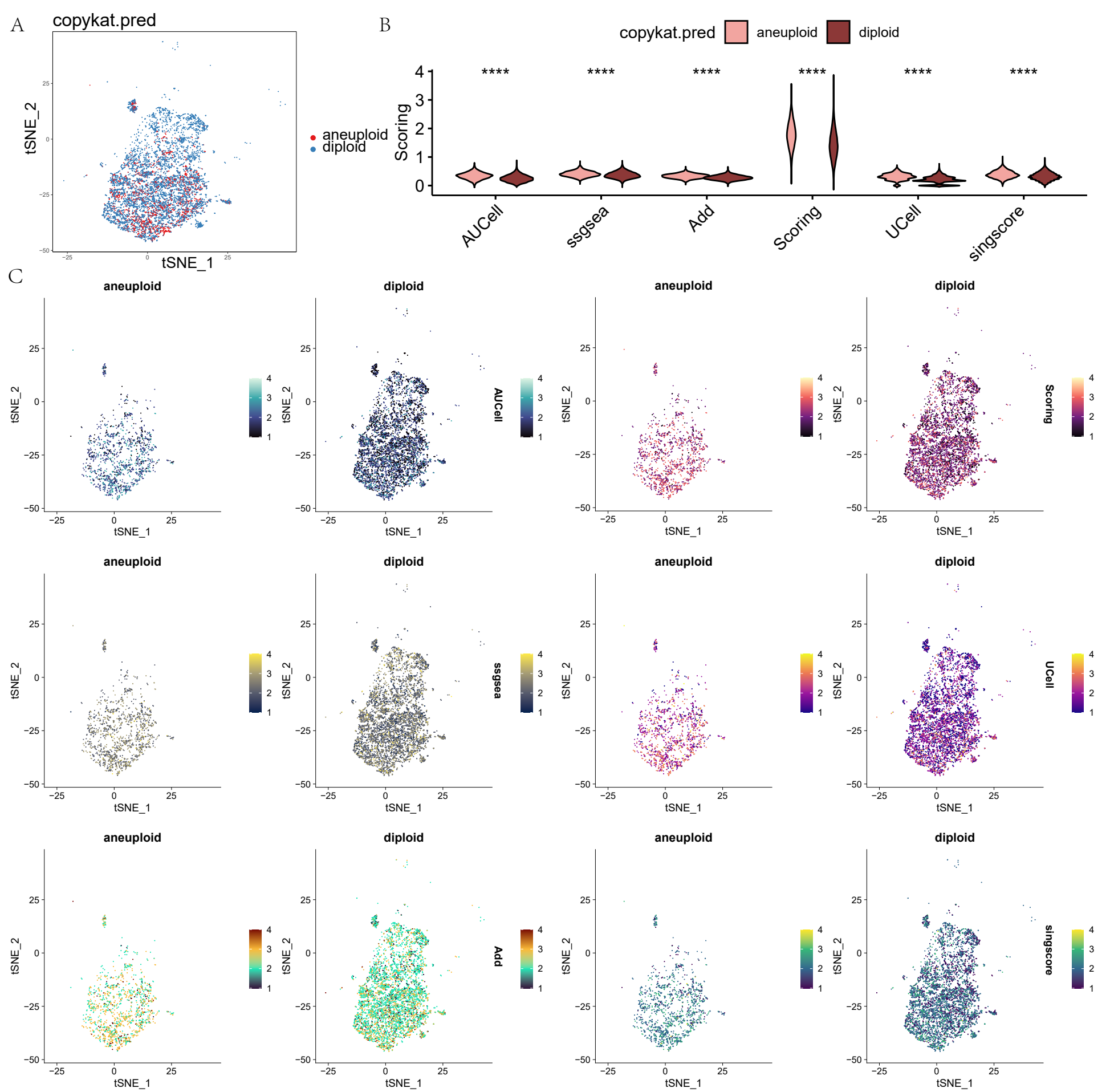

Supplement: Supplementary file 1 [file metabolites-15-00207-s001.zip › Supplementary Figure S2.pdf]

A

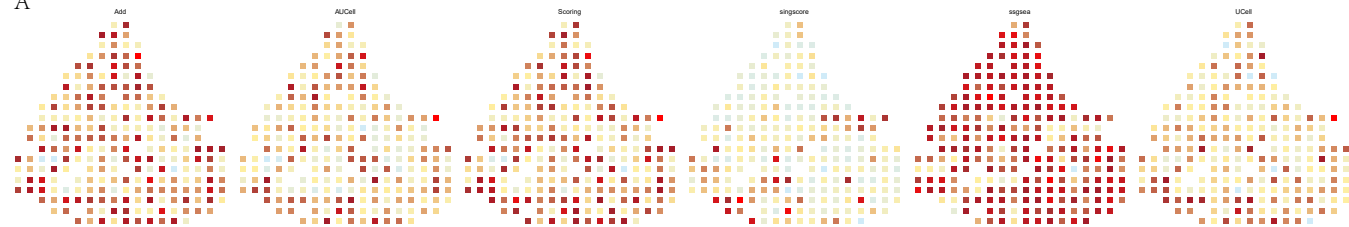

B

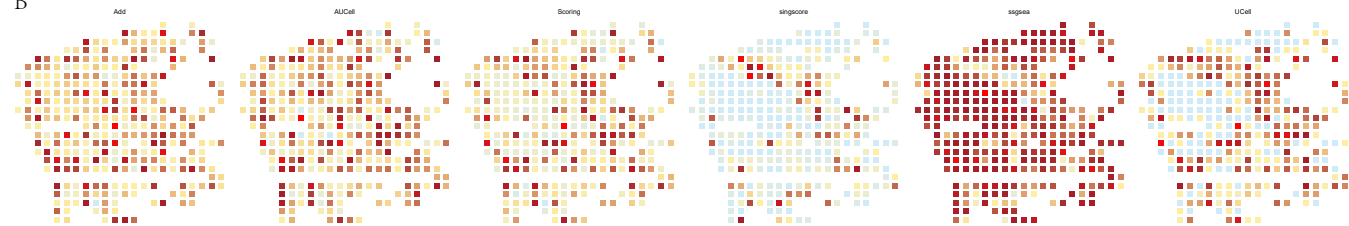

C

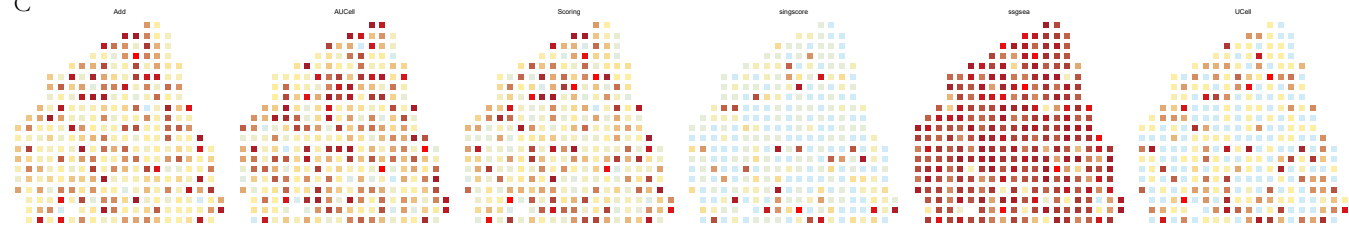

Supplement: Supplementary file 1 [file metabolites-15-00207-s001.zip › Supplementary Figure S3.pdf]

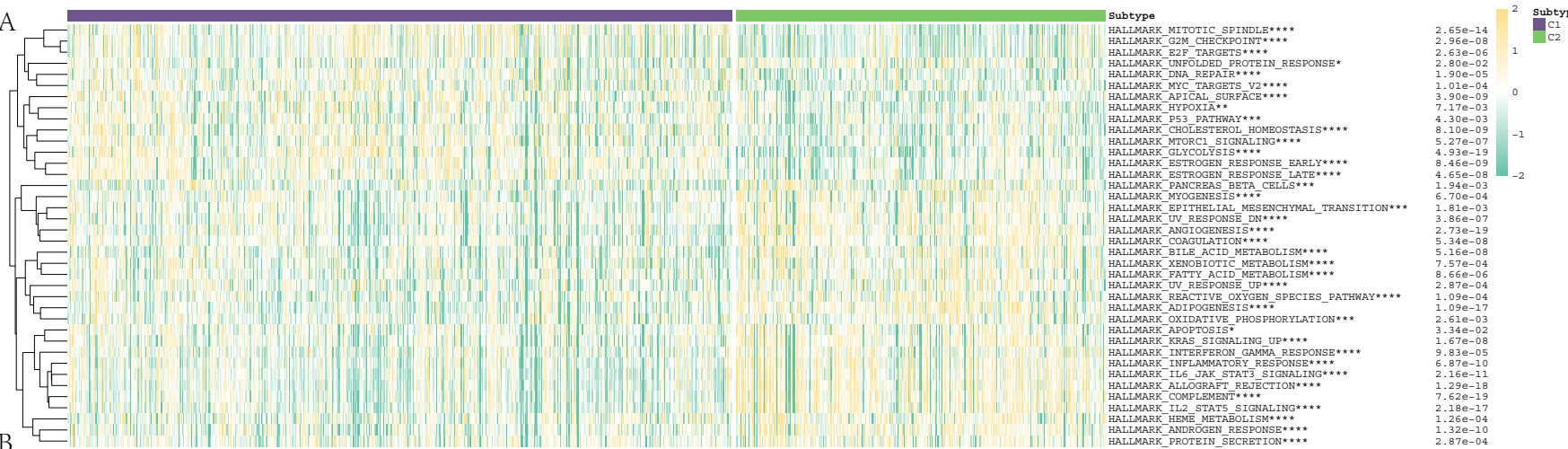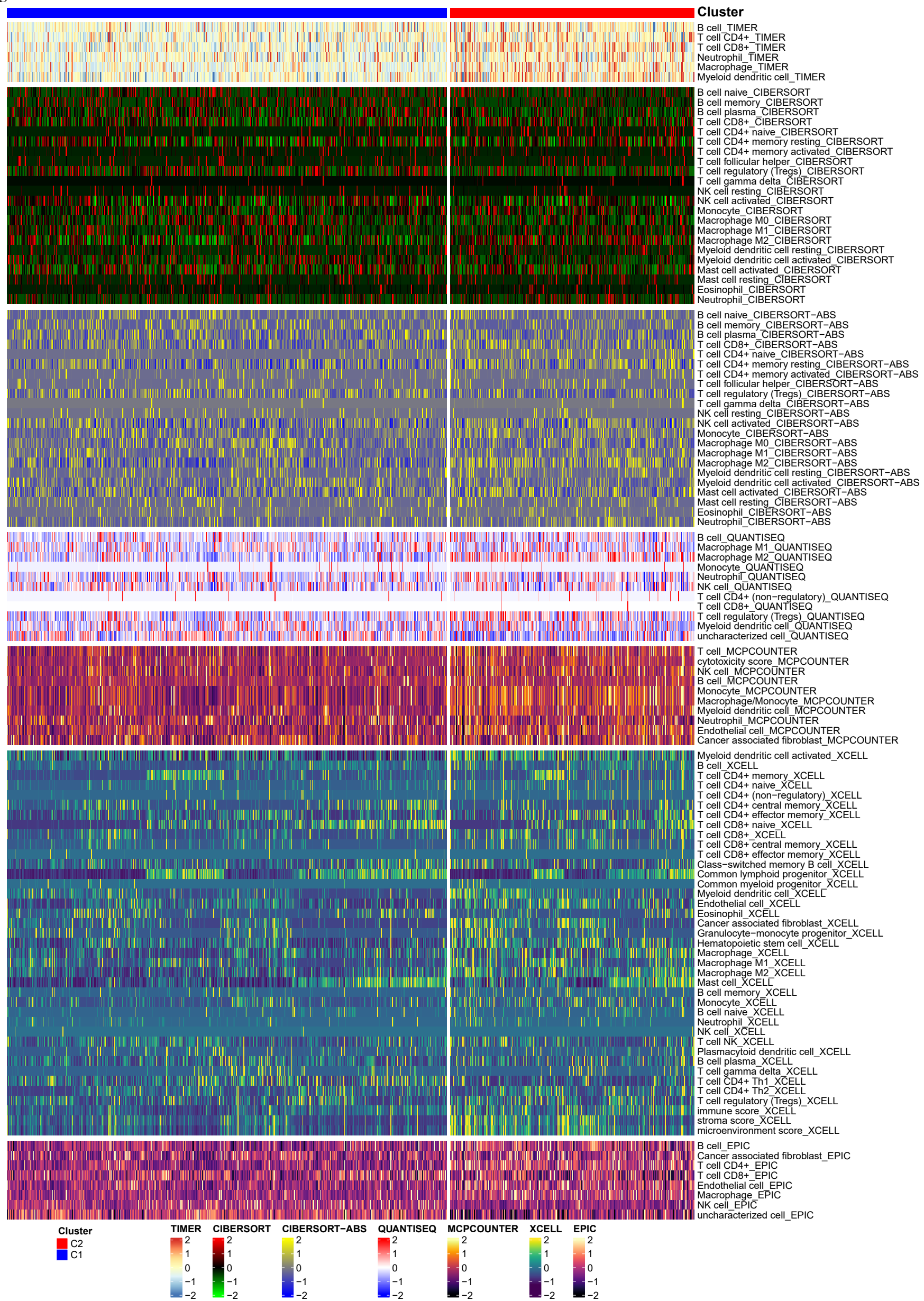

Cluster

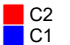

TIMER

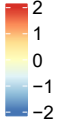

CIBERSORT

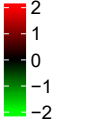

CIBERSORT-ABS

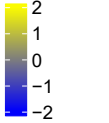

QUANTISEQ

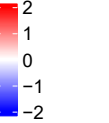

MCPCOUNTER

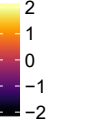

XCELL

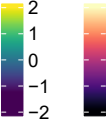

EPIC

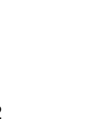

Supplement: Supplementary file 1 [file metabolites-15-00207-s001.zip › Supplementary Figure S4.pdf]
